# Orde ab Chao Method for Disruptive Innovations Creation (With COVID-19 Pandemic Case Application)

**DOI:** 10.3389/fpsyg.2020.581968

**Published:** 2021-02-11

**Authors:** Borut Likar, Denis Trcek

**Affiliations:** ^1^Faculty of Management, University of Primorska, Koper, Slovenia; ^2^Laboratory of E-media, Faculty of Computer and Information Science, University of Ljubljana, Ljubljana, Slovenia

**Keywords:** innovations management, disruptive technologies, forced connections, methodological adaptation, products and services, disruptive innovations

## Abstract

This paper introduces a novel method for the creation of ideas for disruptive innovations. It provides an application of innovation management techniques to specifics of disruptive technologies, which stand behind the Industry 4.0 (and Society 4.0) changes that are taking place at present. Centered around the Ordo ab Chao technique, the paper presents how contemporary disruptive technologies can attain reflections in the complex creative process that has to lead to disruptive ideas and innovations. Quite some innovative thinking techniques already exist. However, they fail to place emphasis on creation of ideas that are tied to emerging disruptive technologies so as to further deploy them in a focused, yet innovative manner. Hence, this paper presents an effective technique that facilitates creation of disruptive ideas with a focused potential for real-life implementations. Practical application of the method related to challenges in higher education processes amid the COVID-19 pandemic is also demonstrated. Based on the understanding of existing disruptive technologies, the technique is used for the adaptation and improvements of distance-learning processes to further add value for students and our society in general. In brief, the Ordo ab Chao technique is a promising tool for systematic development of disruptive solutions, representing a creative synergy between cutting-edge technologies and innovation management approaches.

## Introduction

It is widely recognized that successful disruptive innovations lead to the highest business performance outputs. Based on the work of Christensen (Christensen, 1997; Schmidt and Druehl, 2008; Christensen et al., 2011), a disruptive innovation is defined as something that creates a new value by disrupting the existing value network(s), resulting in displaced dominating market-leading organizations, or dominating products and services. Clearly, such innovations are more often than not generated by newcomers or even complete outsiders, rather than existing market-leading entities. However, the obvious challenge is how to manage disruptive ideas creation processes. Although planning great discoveries and breakthrough ideas is not an easy task, we believe that it is possible to intentionally create conditions that can lead to disruptive solutions. Hence, this paper proposes a new method for achieving disruptive ideas in a “creatively organized” manner. This means that there is a systematically organized base and methodology with simultaneous integration of creative concepts at the core. This is what the *Ordo ab Chao* (i.e., Order from Chaos) method is about—a disruptive ideas creation method.

The paper is structured as follows. In the first section, the *Ordo ab Chao* conceptualization is presented. It starts with the state-of-the-art disruptive technologies properties [e.g., Blockchains (BCs), Internet of Things (IoT)] to clearly understand our starting point. Then, the Forced Connections Technique is presented, which is a well-known creative thinking technique aimed at generating new ideas and solutions to a concrete problem. Following this, the aforementioned disruptive technologies and the technique of Forced Connections are integrated into the *Ordo ab Chao* method that is focused on the development of disruptive ideas for problem solving. In parallel, the action research background of Orde ab Chao is elaborated. In the second section, the development and the application of the method are demonstrated (i.e., distance learning process improvements amid the COVID-19 pandemic). Before the *Conclusions* section, we validate the Orde ab Chao method as a scientific contribution in the field of theory related to disruptive innovations, as well as an applicable tool for practical use. The paper ends with references.

## Overview of the Area

This section first provides background information on technologies, then an overview of relevant existing creative thinking methods followed by COVID pandemic stimulus for this new action research approach that has led to the Orde ab Chao method, described in the third section.

### Understanding Disruptive Technologies

The discovery of the steam machine proved to be an impetus for the First Industrial Revolution. Within it, a few discoveries and business models may be identified (Wiki, 2020), which stand out as crucial driving factors of further developments, e.g., a transition from hand production methods to machines, new chemical manufacturing, iron production processes, potential of water power, development of machine tools, and the rise of mechanized factories. Those researchers and entrepreneurs who were able to find efficient solutions related to these areas had huge prospects for success. Clearly, crucial “elements” of actual technologies have to be identified and applied in a new way to find solutions to current problems and challenges.

When talking about the fourth technological revolution (Industry 4.0), core and enabling technologies, which are driving forces of these developments, can already be identified. Key representatives are BCs, IoT devices, Big Data, Artificial Intelligence (AI), Cloud Computing, Virtual Reality, Additive Technologies, and Security. These key technologies have to be well understood at their core:

• Massive storage and processing are enabled by Cloud Computing, mostly as a result of recent computer communications technologies developments. This way, servers’ farms may be formed anywhere in the internet and offered for deployment where needed as if they were available locally.
• On the other side, the cyberspace is getting densely populated with various small computing devices, IoT, which typically lack computational resources, while mostly performing sensor-like functionalities toward physical world. IoT can be considered as a kind of sensorics layer of the internet, which is becoming the main source of data globally (Gagliordi, 2018).
• Data (including those generated by the IoT) gradually require a storage with a ledger-like functionality that ensures their integrity and reliable provisioning. BCs play a pivotal role here with their deployments of cryptographic mechanisms and consensus protocols, which result in a distributed, incorruptible, and tamper-resistant database.
• Processing of all these data requires appropriate technological means. Storage and processing power described above enable ubiquitous implementations of AI. These run over Big Data and find solutions for various decision-making problems. The more data there are, the better AI solutions become.
• When bridging the above processes, which are mainly taking place in the digital world, toward the physical world, robots and 3D technologies come into play. Clearly, most often their deployment is in advanced tangible output production processes due to the very nature of robots and 3D technologies.
• Overlapping physical and digital realms are virtual and augmented reality technologies that use cyberspace resources to create completely new (virtual) realities or to create outputs that are “implanted” into the physical world and perceived by users as being an integral part of this physical world.
• The systemic “glue” of all the aforementioned technologies is Security Technologies, as without Security (and often also privacy), the above technologies are vulnerable at their core. Their functionalities and functioning can be subverted to an extent that would make them unusable not only for businesses but also for private life deployments.

The main lessons learnt with the aforementioned Industry 4.0 technologies are the following:

• Raw power does make a difference. Many AI principles like (deep) neural networks have been known for a long time, but there has not been sufficient processing power to make them serviceable. This could be referred to as a foreseen disruptive scenario. Many organizations have in fact already been playing with this technology and waiting for its boom to come.
• With the invention of Bitcoin, its BC structure was created as a necessary kind of infrastructure for this digital currency to make it operationally usable. However, it soon turned out that BC can live a life on its own, providing “just” a ledger kind of functionality for numerous purposes not possible so far, including smart contracts. This could be referred to as an unforeseen disruptive scenario. No organization has been playing with this technology, as it was simply unavailable.

### Forced Connections Technique Principles

The purpose of creativity techniques is to avoid established ways of thinking and to find solutions within the known. The longer we deal with one problem, the more stereotypical our thinking is (Likar et al., 2006). Theory of creative thinking encompasses more than a hundred methods, which have different starting points (known vs. unknown problem to participants), goals (useful vs. extremely original ideas), numbers of participants (group vs. individual), ways of performing the creative process (ideas based on creativity of participants, ideas based on solutions from nature, and creativity originating from forced associations), etc. Such techniques are, for example, brainstorming, Gordon’s technique, morphological analysis, bionics, Forced Connections, and others (Likar, 2007). There are also lateral thinking techniques, see e.g., De Bono and Zimbalist (1970). In addition, there are many other approaches and techniques, which can be fruitfully applied for idea creation (Zendejas and Chiasson, 2008; Košmrlj et al., 2015). Due to more and more in-depth knowledge, solutions often become more complex, which is not necessarily effective. The Forced Connections Technique, which is based on Morphological Forced Connections, presented by Koberg and Bagnall in the early 1970s (Putri et al., 2019), is one of those techniques that address the aforementioned problems. Moreover, we can also find “random” combinations, which have led to many discoveries in the past, e.g., vulcanization process, or discovery of penicillin.

Hence, the Forced Connections Technique is based on our ability to generate associations between disparate items, e.g., constructs, ideas, pictures, physical objects, or words. Its aim is to make a link between problems and challenges on one side, and randomly selected words and constructions on the other. It relies on random external triggers that force people to make a connection between the problem at hand and the triggers, which cause people to broaden their perspective and thus create original ideas that can represent the base for disruptive ones. The first step is to find random words. We look for them in dictionaries, lexicons, professional books, indexes, and suchlike. It is important not to choose only the words that we find interesting—they have to be chosen randomly. In its simplest form, we can place our finger on an index, find the word, and write it down on a paper.

The Forced Connections Technique addresses four types of connections. However, in a case where we apply it to a concrete problem, i.e., our challenge, and look for new solutions, we have two basic possibilities presented in Table 1.

**TABLE 1:** Typology of connections (associations) within Forced Connections Technique.

Connections	Random word—a concrete problem
**Direct**	A
**Indirect**	B

By connecting a random word with a concrete problem, solutions may be found beyond known frameworks. One such example is the car rust problem. Random words can be window, plastic, water, and crocodile. To begin with, the conjunction “and” is omitted from the chosen words.

The direct link method (cell A in Table 1) gives the following answers:

• Window: A car should have more windows to minimize rust.
• Plastic: A car should be made of plastic.
• Plastic: A car is plasticized or made of plastic.
• Water: We have no direct association.
• Crocodile: We have no direct association.

The indirect link (cell B in Table 1) offers several options:

• Window: We use a computer with MS Windows to make it easier to solve the problem of rust, so we do a computer simulation of rust.
• Plastic: We look for some kind of plastic with mechanical properties of steel.
• Water: It causes rusting, but it is facilitated by chemical elements found in the atmosphere and thus air pollution should be reduced air pollution.
• Crocodile: It lives in water with various fish like electric stingray. Continuing with electricity, we come to cathodic protection, which nowadays successfully protects cars’ metal parts, bridge structures, etc.

The most important “asset” of the idea creation process is a team—building an appropriate team is one of the key success factors. There are many useful concepts and methodologies, e.g., the VICTORY model proposed by Tang (2019), which addresses non-cognitive, cognitive, and environmental enablers of team creativity.

### Distance Learning Challenges

The development of a method for creation of ideas for disruptive innovations follows. Using a pilot study, it will be applied to current pandemic distance learning challenges. Therefore, this section deals with theoretical background related to distance learning.

We start with a wider picture related to adherence of educational systems and the needs of societies and economies in 2010. So, as early as (OECD, 2010) recognized that these systems must adapt to the rapid development of the economy and enable young people to develop skills that will help them be as effective as possible at their work (Belak et al., 2017). The society and all its crucial sub-structures (economy, educational system, R&D sphere, medical system, culture, and governmental institutions) are driven by people. Besides, future development is based on new generations, so the education system is the most important pillar of their knowledge, experiences, and personal competences. Consequently, this system is crucial for our societies and countries in general. The obvious question is whether, in its current form, it is really aligned with the real needs. Sir K. Robinson, one of the best-known thinkers in the education sphere, has stressed many times that this system has never been seriously modified, while other areas of society, e.g., economy, culture, and personal development, have made a huge progress in the last 50 years (Robinson, 2010). The opinion of Carmody (2009), co-author of the bestseller *Disruptive Class: How Disruptive Innovation Will Change the Way the World Learns*, supports this thesis. He explains that the current form of teaching “is unable to provide today’s pupils with the skills they need to master in order to interact with and within the digital society”. He highlights the need for a disruptive education that approaches learning in a somewhat different way.

During the COVID-19 outbreak, UNESCO defined a set of actual challenges (UNESCO, 2020). Those that are related *to our challenge, which is to make distance teaching process comparable to face-to-face lecturing*, are given below:

• Interrupted learning: Schooling provides essential learning, and when schools close, children and youth are deprived of opportunities for growth and development. The disadvantages are disproportionate for under-privileged learners who tend to have fewer educational opportunities beyond school.
• Unequal access to digital learning portals: Lack of access to technology or good internet connectivity is an obstacle to continuous learning process, especially for students from disadvantaged families.
• Social isolation: Schools are hubs of social activity and human interaction. When schools are closed, many children and youth miss out of on social contact that is essential to learning and development.

UNESCO suggests some distance learning solutions like digital learning management systems, external repositories of distance learning solutions, systems built for use with basic mobile phones (or with strong offline functionality), Massive Open Online Course Platforms, self-directed learning content, mobile reading applications, collaboration platforms with live video, and tools for teachers to create digital learning content.

McKinsey’s study, although focused exclusively on higher education, showed similar results, with an additional important message. Universities and colleges are expected to be under pressure to develop and deliver online courses, which will put their budgets under even more pressure. Online programs have traditionally been cheaper (Bevins et al., 2020). However, this financial pressure threatens to significantly reduce the quality of such kind of lecturing. The authors’ own experiences show that virtual lecturing can be rather effective even if “only” traditional methods are adapted. However, it is important that there is a real-time contact between a lecturer and students. It seems that what counts are the following three elements: interactivity, interactivity, and interactivity. On top of this, even the most influential and dominant massive online course platforms like Coursera have a success rate well below 10% (i.e., those who finish a course). So, the second goal is *to drive the developments in higher education sector into personalized education.*

Summing up, amid new COVID-19 reality, new models and solutions to push traditional methods to comparable levels as in ordinary settings are needed. Further, we should aim also at new ways to additionally add value and deliver enhanced education for new generations at acceptably increased costs through personalization of education processes.

### Action Research Background

According to Reason and Bradbury (2008), the primary purpose of action research is to produce practical knowledge that is useful to people in the everyday conduct of their lives. Therefore, action research is about working toward practical outcomes and it is also about “creating new forms of understanding”. As stressed by Koshy et al. (2010), action research creates knowledge based on enquiries conducted within specific and often practical contexts. The purpose of action research is to learn through action that then leads to personal or professional development. The spiral model gives an opportunity to tackle a phenomenon at a higher level each time and so to progress toward a greater overall understanding.

The main idea behind the development of the Orde ab Chao method is to enable (and to enhance the probability of) producing disruptive ideas. It represents a new knowledge, with a clear practical outcome. Disruptive ideas and consequentially innovations are highly appreciated within the innovation typology, yet very difficult to create. When the basic concept of the Orde ab Chao method has been developed, we conducted a research focused on its verification and further development. We used an action research model employed by Elliot (1991 :71), which includes identification of a general idea, reconnaissance (fact-finding), planning, action, evaluation, amending plan, and taking a second action step.

## Orde AB Chao Action Research Methodology

In this section, the crucial elements of action research methodology behind the Orde ab Chao method are presented, while the section *COVID-19 Pandemic Application in Higher Education* addresses concrete steps related to this methodology.

We applied a simplified methodology presented by Urquhart et al. (2012), which addresses various holistic aspects of research—for our methodology, the potentially disruptive solutions present the most important criterion. In addition, topics of fluency, i.e., number of ideas and their originality (Dixon, 1979; Ding et al., 2014), potential benefits for users (and also creative session participants’ structure), and feedback from participants and from moderators, are covered as well. By doing so, we addressed some important issues in a structured way. At the conceptual level, the approach is similar to cancer treatment-related approach described by de Baca et al. (2015), where authors addressed the following: patient demographics; disease, diagnosis, and prognosis; tumor board dispositions and decisions; graphic timeline; pre-resection workup and therapy; resection workup; interpretative comment summarizing pertinent findings; biobanking data; postresection workup; and disease and patient status at follow-up. Furthermore, we wanted to create a methodology in a way that would enable us to observe, measure, and record some important properties of the *Ordo ab Chao* method. Such a concept also represents a step toward synoptic reports. Synoptic reports utilize a standardized template to record data and have emerged as an alternative to narrative reports. They have a higher degree of overall completeness compared to narrative reports: 60% vs. 45% (Eng et al., 2018). In this way, our methodology may be tested also by using other glossary databases and finding the most suitable databases related with a certain challenge.

Regarding evaluation of ideas created, there are various approaches addressing different dimensions. Dean et al. (2005) addresses novelty, workability, relevance, and specificity. A similar approach can be observed by Kudrowitz and Wallace (2013) that focuses on creativity, novelty, usefulness, product–product worthiness, and clarity. Some authors also add usefulness of the answer, with which we check how useful it is in a certain situation (Pečjak, 2001). Correa and De Moura Ferreira Danilevicz (2015) focus more on aspects related to company’s strategy, feasibility, financial aspects, and others. With our research, which is not yet focused on a concrete company’s strategy, we decided to evaluate two dimensions of ideas: originality and potential benefit for users.

The obvious question is why we have decided for these two criteria. Originality is a precondition for breakthrough ideas/innovations (Christensen et al., 2015). Only original ideas represent a clear distinction to already known solutions and have the potential to be disruptive. In addition, only original ideas are needed; however, they must also have potential to develop into innovations. Therefore, the next criterion is related to potential benefit for the user.

To assess originality from a qualitative point of view, the scale of Dean et al. (2005) was applied, deployed in their research on the evaluation of ideas as follows:

• 4—The idea is rare, unusual, imaginative, resourceful, and surprising; it can even be humorous.
• 3—The idea is unusual; it shows some imagination.
• 2—The idea is interesting.
• 1—The idea is ordinary, boring.

To access the potential benefit for a user, Kano’s concept (Chen and Chuang, 2008; Mikulić and Prebežac, 2011; Yang and Yang, 2011) was applied.

• The must-be or basic quality: At this point, customers become dissatisfied when the performance of this product criterion is low or the product attribute is absent (such as a bicycle breaks). However, customer satisfaction does not rise above neutral with a high-performance product criterion.
• One-dimensional or performance quality: Here, customer satisfaction is a linear function of a product criterion performance. High attribute performance leads to high customer satisfaction and vice versa.
• The attractive or excitement quality: Here, customer satisfaction increases super linearly with increasing attribute performance. There is not, however, a corresponding decrease in customer satisfaction with a decrease in criterion performance (Chen and Chuang, 2008).

It is better to perform the evaluation process with a larger number of evaluators and not just one, as this ensures greater credibility and objectivity of the evaluation of ideas. Besides, most of them should not be a member of the creative team due to objectivity. It is also essential that experts for this phase are familiar with state-of-the-art solutions and have appropriate knowledge to evaluate various aspect of ideas (originality, benefits for users, etc.).

### Ordo ab Chao Method

This method is based on two inputs, the Forced Connections Technique and disruptive technologies related to selected words, also referred to as *elements*. It consists of the following steps:

1. *Problem definition.* First, the problem/challenge is narrowed down and clearly defined.
2. *Creation of disruptive technologies elements database*. Instead of using random words for creating unexpected ideas, keywords from the domain of the presented eight kinds of disruptive technologies (BCs, IoT devices, Big Data, AI, Cloud Computing, Virtual Reality, Additive Technologies, and Security) are used. With this important step, the state-of-the-art disruptive technologies into our problem-solving process is applied. For each of these technologies, appropriate *elements* are sought. For example, for Virtual Reality, an appropriate source including crucial *elements* needs to be found. This can be appropriate glossary in the area of Virtual Reality. Consistent with our experience, it is very useful if the glossary has the following characteristics:

• it includes state-of-the-art words that are narrowed down into the final set of elements;
• it is comprehensive enough, yet not excessively long (roughly 50 elements are suggested);
• each element has a short description, so that also a generalist can understand the meaning and use it in the creative session.

3. *Creative process* (implementation of Forced Connections). For the starting problem, a mental process looking for creative association is performed (direct or indirect), which represents a possible original solution of the defined problem.

• Direct: either technological solutions, which are based on elements for each of the technology used can be expected, or
• Indirect: Other types of ides may also be expected, e.g., organizational with no direct connection with the disruptive element.

On one side, a highly creative and often chaotic brain process takes place, while systematically following the identified elements. A two-phase approach is suggested:

• Each participant chooses his/her own elements for one technology and goes through possible associations. This can take 30 to 60 min not to exhaust the participant, and to get a pool of independent ideas.
• At a joint session, participants present their ideas and, with brainstorming, upgrade these ideas using the group dynamics. The aim is to upgrade their ideas with new suggestions and also to focus them on concrete problems where applicable.

4. *Finalization*. This is a final phase that is aimed at:

• Making a selection of most original ideas with the highest potential.
• Merging similar ideas. It often happens that ideas are overlapping, so it makes sense to merge them (overlapping ideas trigger similar or same solutions).
• Merging ideas that address the same problem. This streamlines the diversity of generated solutions into corresponding groups.

This phase, i.e., selection of ideas, was done by a group of four people; two of them were members of creative session (step 3) and two were new and therefore more objective. The selection process followed two criteria:

• Originality of an idea was measured with a scale suggested by Dean et al. (2005) as presented in Literature review: Evaluation of results. As presented, a score of 4 is the highest (rare, unusual ideas, while 1 means ordinary, boring ideas).
• The potential benefit for the user was measured with three categories as presented in Literature review (1 = basic quality, 2 = performance quality, and 3 = excitement quality).

Following the action research methodology, we also suggest guidelines for application in praxis in the section *Practical hints for Ordo ab Chao users*.

## COVID-19 Pandemic Application in Higher Education

In this section, an application of the *Ordo ab Chao* method for addressing challenges in education processes amid new worldwide reality caused by the COVID-19 virus is presented.

For the Orde ab Chao method, a pilot study was performed to test if it provides expected disruptive results, to examine the structure of participants teams related to their background knowledge, to observe the number of achieved ideas, and to evaluate their originality and potential related to expected user’s experience. In addition, the feedback of participants and the moderator can provide constructive information.

Seven groups of participants, each consisting of approximately three participants, were formed. In two of the groups, the participants with mainly technical competencies (hereinafter referred to as technical team) were included. Two groups included participants with mainly social science competences (hereinafter referred to as social team), while three groups consisted of participants with blended competences (hereinafter referred to as blended team), namely, two to three participants with social science competences and approximately one member with technical competencies. Each group used one glossary element set, either IoT or VR. Besides, there was a coordinator (an expert in the Orde ab Chao methodology and glossary element support) that was of assistance to the participants.

**FIGURE 1:**
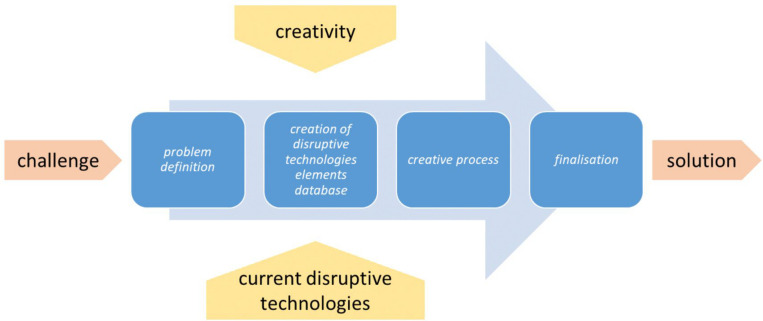
Ordo ab Chao method structure and steps.

Before the creative session (please see Figure 1), step 1 (Problem definition) and step 2 (Creation of disruptive technologies elements database) were already done by the authors. Otherwise, it should be done by the coordinator.

The duration of step 3 (Creative process) was approximately 15 min for presentation of the method and another 15 min for preparing for work in the Pilot Creative Session, where participants started working by themselves and the coordinator helped them to completely understand the method and their task. The pure creative session related to association took approximately 60 min.

For step 4 (Finalization), additional explanation is needed as to the selection of most original ideas. Following the Orde ab Chao concept, we wanted to achieve original ideas with the potential to become disruptive and/or breakthrough innovations. Therefore, all the ideas were evaluated following the two criteria:

• originality from a qualitative point of view (Dean’s criteria 1–4; 1—The idea is ordinary… and 4—The idea is rare, unusual…),
• potential benefit for the user (Kano’s concept: 1—basic, 2—performance, 3—excitement).

The evaluation process was performed after the creative session and took approximately 60 min (per group).

### Problem Definition (Step 1)

Based on the described distance learning challenges, the research problem/challenge was defined, i.e., how to develop new models and solutions to push traditional methods to the same levels as in ordinary settings (goal I). Further, we should also aim at new ways to additionally add value and deliver better education for new generations at acceptably increased costs through personalization of education processes (goal II).

### Creation of Disruptive Technology *Elements* Database (Step 2)

The mentioned six categories of Industry 4.0 disruptive technologies are addressed: BCs, IoT devices, Big Data, AI, Cloud Computing, Virtual Reality, Additive Technologies, and Security. For each of these six technologies, appropriate *elements* first need to be found. For the purpose of demonstration, emphasis is placed on two categories: Virtual Reality and IoT. For performing this step, two dictionaries were chosen and the terms below were obtained:

• Virtual Reality (VR) glossary encompassed 61 terms, namely (Simpson, 2020): *360, Experience*, *360 Live Streaming*, *360 Panorama*, *360 Photo*, *360 Video*, *3D Audio*, *All-In-One, Headset, ARCORE, ARKIT, Augmented Reality*, *Avatar*, *Cave Automatic Virtual, Environment*, *Computer Generated Virtual Reality*, *Computer Aided Design*, *Data/Wired Glove*, *Duck Test*, *Experiencer/User/Player*, *Extended Reality*, *Eye Tracking*, *Field Of, View*, *Foveated Rendering*, *Gaze-Activated Content*, *Gl Transmission Format*, *HAPTICS*, *Head, Mounted Display/Headset/Goggles*, *Head Tracking*, *Head-Up Display*, *Heatmap*, *Hotspot*, *HTC VIVE*, *Immersion*, *Immersive Reality*, *Inertial Measurement Unit*, *Latency*, *Light, Field Technology*, *Locomotion*, *Mesh*, *Mixed Reality*, *Mobile Headset, Oculus Rift*, *Perambulation (Locomotion)*, *Positional Sensor*, *Presence*, *Real Life*, *Reticle*, *Room-Scale*, *Six, Degrees-Of-Freedom*, *Spatial Mapping*, *Spherical Panorama*, *Stitching*, *Surface Detection*, *Teleportation, Telestration*, *Tethered Headset*, *Transportation*, *Unity, 3D*, *Virtual Reality (VR)*, *Virtual Reality Sickness*, *VR Marketing*, *Vuforia*, *WebVR.*

• IoT Glossary encompassed 55 terms, namely (VR, 2020): *Actuator, Advanced Message Queuing Protocol, Application Agents, Bluetooth Low Energy, Chirps, Competing Consumers, Connected Devices, Connectivity Protection, Constrained, Application Protocol, Data Filtration, Device-Agnostic Control, Direct Messaging, Edge Gateway, Edge Layer, Embedded Device/Systems, Endpoint Device, Flow-Based Programming, Geofencing*, *Haze Computing, Home Automation, iBeacon, Industrial Internet, Integrator, Internet of Things*, *Internet Protocol Suite*, *IoT Cloud Platform, IoT Development Board, Lightweight Protocol, Long Range Communication Protocols, Low-Power Devices, Machine-to-Machine, Mesh Network, Microcontroller, Messaging Protocols, Message Queuing, Telemetry Transport, Multi-Agent System, Near-Field Communication, Operability, Personal Area Network, Propagator, Radio Frequency Identification, Real-Time Operating, System, Releasability, Sensor, Sensor Network, Single-Board Computer, Site-Level, Management, Store and Forward, System on a Chip, Transmission Control Protocol/Internet Protocol, Ubiquitous Computing, Wearables, Wi-Fi, ZigBee, Z-Wave.*

The above two dictionaries have also been used for description and better understanding of the *elements*.

### Creative Process and Finalization (Steps 3 and 4)

Due to too many possible associations, and not to extend the length of the paper, only those ideas with the highest potential are partially merged together and presented (steps 3 and 4 of the presented method are merged).

#### Glossary Element: VR—

Hotspot, Avatar—for a Detailed Explanation of Element, See IoT (2020) and Simpson (2020)

#### Problem

A class discussion is extremely important for a successful education process. However, there are relatively few verbal questions from students when using distance learning tools. Students seem to be more reserved to expose themselves by raising questions. So, typing in a chat window is often preferred, which is mainly done impulsively. Consequently, a question is often not well articulated, also because of required extensive typing performed in a short period of time.

#### From an idea to solution

The idea is that students connect to an artificial agent anonymously. This agent leads students to articulate their questions well, and once their question is well articulated, the lecturer is stopped and the agent tells the question to the whole virtual audience. In addition, the chat channel can be proxied to enable anonymity, when such articulation can be sacrificed, or is not so important.

#### Glossary Element: VR—

Eye Tracking, Field of View, Head Tracking, Heatmap, Latency. Additional Word, Pupils Dilation

#### Problem

Problem of “talking to the wall”. During distance lecturing, one of the crucial problems is that a lecturer has an impaired “real-life” feedback from students compared to face-to-face lectures. Nevertheless, this feedback is important, as it enables good lecturers to immediately react. Practical experiences show that, with distance learning, students often lose concentration and may also engage in other activities (e.g., browsing web pages, texting, and having all kinds of other distractions in their own environments). For a lecturer, it is almost impossible to check faces of tens or hundreds of students to get visual feedback on their concentration, not to mention that such feedback on face-to-face lecturing includes also body language. In addition to limited camera captured area, online lecturing also often provides poor quality video, causing additional problems.

#### From an idea to solution

The telemetry is used to analyze the data gathered by the student’s computer and to give feedback to the lecturer; namely, which student has lost concentration or is not following the lecture. This information would be shown as a colored bar (going from red to green) in the (sub)window of each participant and would enable the lecturer to react accordingly. Certainly, this should not be an element that leads to a “punishment”, but a sign to include additional dynamics, start a discussion, suggest a break, and suchlike. The base for telemetry can also be smart glasses with sensors for eye tracking, field of view analysis, head tracking, and dilation of the pupil. Additional sensors may be placed on glass holders so as to measure skin conductance, oxygenation levels, etc. As these types of information raise privacy issues, an appropriate intermediate service (like a proxy-avatar) can regulate this info, so only the aforementioned color bar indicator is obtained in each student’s sub-window, while the rest of the data are destroyed in real time. This way, privacy is preserved.

#### Glossary Element: VR—

Environment, Augmented Reality

#### Problem

Video platforms are technically well prepared, yet participants do not have the same feeling as in the classroom.

#### From an idea to solution

Participants’ photos are not presented in separated frames but are elements in a virtual classroom. It can be done in a simple or a more sophisticated way, using the Augmented Reality principles.

#### Glossary Element: VR—

Experiencer/User/Player

#### Problem

Students are often less motivated to cooperate in comparison with the classical classroom (they report the energy in the class is different). Therefore, additional elements of motivation should be applied.

#### From an idea to solution

Students get bonus points for their active cooperation within the discussion, e.g., time of discussion. An additional element is optional, i.e., that a lecturer after the students’ discussion “weights” the relevance of student’s discussion.

#### Glossary Element: VR—

Head Tracking

#### Problem

Presenters do not have the impression of the whole auditory and their agreement/disagreement with the content in case of many participants or if they do not want to share their photo on video platform.

#### From an idea to solution

Detecting students either nodding/shaking their heads, and as a next step, the system would send the lecturer only aggregate information from the audience (or individual) about non-verbal communication.

#### Glossary Element: VR—

Heatmap, Head Tracking, Eye Tracking

#### Problem

When the lecturer asks the audience to read the text, he/she does not know when students finished reading.

#### From an idea to solution

The computer video system recognizes eye/face movement patterns when participants are reading. Identifying how many people have already read the text (via Heatmap, Head Tracking, or Eye Tracking), a professor receives the info on percentage of those who have already finished. Thus, he/she can move the text forward.

### Action Research Methodology Implementation

In the previous section, the basics for action research were presented. As it will be explained, the whole method was being developed step by step, cycle by cycle. These cycles, following the action research paradigm, will be presented as follows: reconnaissance (fact-finding), planning, action, evaluation, measuring/evaluating results (ideas), feedback from moderator (observation) and feedback from participants, and amending plan.

#### Cycle 1

The starting point is based on literature review and authors’ ideas. First, we selected participants (technical team) for the Orde ab Chao creative session. They received brief instructions regarding methodology. In this cycle, there was no moderator. At the end of the creative session, we provided evaluation of ideas (independent team of two to three people) and prepared a brief open question interview for the participants of the technical team. The first group (one technical team) performed the Orde ab Chao method. A creative process was based on two glossary element sets. During the evaluation phase, evaluators assessed ideas—the only criterion was their disruptive potential. Based on the interview with the team, we realized that brief instructions were not sufficient and part of created ideas was not clearly focused into searching solutions of the basic challenge. The evaluators also reported that ideas were “very technical”. There was also a remark that the number of ideas was quite low compared to other creativity techniques, e.g., brainstorming or Forced Connections, where 30–50 or more ideas can be expected (Likar, 2007). Based on these experiences, we prepared an improved methodology for Cycle 2. It included more detailed personal instructions for creative session implementation. In addition, a more detailed and comprehensive evaluation methodology was introduced (see the section “ORDE AB CHAO ACTION RESEARCH METHODOLOGY”). We also wanted to test how another structure of participants—not only technical team—would perform. We decided to test a social sciences team and a technical team.

#### Cycle 2

Based on the amending plan, we performed the second cycle with social teams (two groups) and a technical team (one group). When all the Orde ab Chao steps were done, evaluators did their work following the more detailed and comprehensive evaluation methodology already presented in section Orde ab Chao conceptualization and action research evaluation methodology. First, we evaluated the creative session results: number of ideas, their originality, and potential benefits for users. We also performed interviews with participants. The observations showed some interesting differences in results between both groups. Social groups had more ideas, but originality and potential benefit for users seemed to be lower. The interviews showed that participants who were familiar with the Forced connection method had no problems. Yet, the other group faced problems, especially in developing indirect connections (see Table 1). In addition, we realized that with the present quantitative (number/evaluation of ideas) and qualitative instruments (interview with team members), we do not have a sufficient insight into the creative session of the teams. Based on these experiences, we prepared additional improvements in the method. We first upgraded the introduction of the Pilot Creative Session. After the ex-cathedra presentation, participants started working by themselves and the coordinator helped them to completely understand the method and their task. The coordinator was also a part of the method and participated in the creative session. Though we developed a system, which was composed of humans engaged in interaction, using gestures and language resulted in the creation of impressions and the transmission of information as suggested by Koshy et al. (2010). In addition, we upgraded the evaluation toolkit with an interview with the coordinator and received an insight into the Orde ab Chao process. We also wanted to test the idea of blended teams, including participants with social and technical skills.

#### Cycle 3

Based on the amending plan, we performed the third cycle with social teams (three groups). There was a coordinator, who firstly performed a Pilot Creative Session, supported the session, and also observed the work. In addition, we collected participants’ as well as moderators’ feedback and evaluated ideas following the already presented methodology. The results of this cycle in comparison with previous cycles are presented in the next subchapter.

### Overall Evaluation of Results

As mentioned, we had seven groups, which is obviously not enough for quantitative analysis. Therefore, the focus was mainly on qualitative results. Quantitative results were gathered only as indicators for further research, the main one being that each group created approximately 19 ideas (average = 18.6).

As to the rest of the criteria, the first impression is surprising. Regarding originality, 20% of them had the highest ranking 4, and regarding the criteria of potential benefit for the user, 10% of all ideas achieved the highest grade (excitement). Some differences among groups were also detected. The social team and blended team had comparable number of all ideas, while the technical team had less. However, what is more interesting is the number of most original ideas, which was approximately two times higher in the blended team and the technical team, compared to the social team. Even more fascinating is the number of “excited” ideas (potential benefit for the user criterium). The blended and technical teams had six to seven times more ideas in the mentioned “excitement” rank. When analyzing the session and the results, it was concluded that the technical team understands the glossary elements and technical solutions better. It seems that they are more critical to their own ideas (self-criticism), which is one of the problems of creativity sessions, especially by experts (Pečjak, 2001). However, at the same time, their ideas seem to be more realistic. Basic average values are presented in Table 2.

**TABLE 2:** Indicative results—number of ideas.

NUMBER OF IDEAS	All seven groups	Social team	Blended team	Technical team
All ideas	19	17.0	20.3	12.5
Originality = 4 (rare)	3.6	2.0	4.3	4.0
Potential benefit for the user = 3 (excitement)	2.4	0.5	3.0	3.5

#### Evaluation of Creative Sessions

Further evaluation included feedbacks from participants and from moderators that are included in the subsection below.

##### Participants’ feedback

• At times, it was not easy to look for associations to glossary elements that we did not understand well.
• We find the method very interesting and was helpful in creating ideas on the subject.
• The method is useful. It helps with wider problems.
• The method somewhat limits us to find solutions from one area (technology). If we did not use it, we could find another solution from another area.
• Some of the ideas are very original to us—without this technique, we would not have come up with them.
• The task seemed very instructive to us, as we were thinking for the first time about what is not good with the distance learning platform (e.g., Zoom) and how to improve it.
• A lot of cooperation was needed.

##### Moderators’ feedback

• The methodology of the work needs to be explained first in the plenary session. Occasionally, some participants only looked for associations, but they were not focused on finding a solution to the initial challenge. It makes sense to conduct a Pilot Creative Session (3–5 min) in each group, where participants practically start working according to the methodology, and the moderator guides them while ensuring full understanding and proper implementation of the concept.
• As the participants (mainly social science participants) are often not familiar with technical glossary elements, their understanding is important for efficient idea creation process. Therefore, we suggest to create blended teams—a combination of participants with social science background and technicians, who are familiar with technical glossary terms. If such a person leads the creative session, he/she can also manage and direct the process and step by step toward original, but also realistic solutions.
• After 30–45 min of intensive creative work, participants’ creativity decreased. Therefore, if 45 min is not enough for the whole session, we suggest to make a break and then to continue.

### Practical Hints for Ordo ab Chao Users

Although the Ordo ab Chao method can be run individually, group dynamics plays an important role in brainstorming. To run it effectively in a group setting, it is recommended to do as follows:

• Before starting work in groups, it makes sense to conduct a Pilot Creative Session (3–5 min).
• Define the problem you want to solve in a crystal-clear way to all participants.
• Keep the session focused on the problem solving.
• Ensure that no one criticizes or evaluates ideas during the session. Criticism induces a component of risk for group members when putting forward an idea. This stifles creativity as well as most original ideas and cripples the free-running nature of a good brainstorming session.
• Encourage an enthusiastic, uncritical attitude among members of the group. Try to get everyone to contribute and develop ideas, including quiet members of the group.
• Let people have fun while brainstorming (encourage them to come up with as many ideas as possible, from solidly practical to wildly impractical ones, welcome creativity).
• Ensure that no “train of thoughts” is followed for too long.
• Encourage people to develop other people’s ideas, or to use other ideas to create new ones.
• Try to keep the process running smooth and making it fast enough so it does not exceed 60 min when people typically get exhausted.
• In order to find a complete solution, it would make sense to use another technique that does not guide thinking, for example, brainstorming.
• If 45 min is not enough for the whole session, it is suggested to take a break before continuing.

It is also important to select and invite appropriate participants. Based on literature (e.g., Likar, 2007) and evaluation of our pilot study, we suggest the following structure of participants (Table 3). In addition, the VICTORY model elements can be useful (Tang, 2019) in synthesizing both non-cognitive (vision, openness, risk-taking, yes-I-can mindset) and cognitive (ideation, combination) antecedents of team creativity.

**TABLE 3:** Optimal characteristics of participants.

Step	Structure	Type of competencies
*1—Problem definition*	Coordinator (in cooperation of the problem owner)	Person who understands the challenge, is familiar with technology and creativity management
*2—Creation of disruptive technologies elements database*	Coordinator	Person who understands the challenge, is familiar with technology and creativity management
*3—Creative process*	Blended team (social science, technical science background—at least one)	Creative persons with various experiences
*4—Finalization*	Mixed team—1–2 participants of the Creative process, the other should be new and therefore more objective	Experts familiar with state-of-the-art solutions and have appropriate knowledge to evaluate various aspect of ideas (originality, benefits for users, implementability, etc.).

## Conclusion

The current worldwide pandemic has revealed the need for many existing services to be adapted to a new normal, or even to introduce new ones, based on new paradigms. One such notable case is a higher education sector.

This situation and gained experiences in distance learning lecturing at the university level triggered the authors of this paper to look for solutions. Being specialized in innovation management techniques, it was found that there is no such technique, which would focus on a completely “disruptive scenario”. Actually, the current pandemic is a disruptive scenario itself, while recent Industry 4.0 (and society 4.0 in general) disruptive technologies are already a fact. Yet, there is a missing link that would provide “disruptive scenarios and technologies” focused innovation processes. Such an approach would represent an important novelty compared to existing methods for idea creation.

Therefore, this paper presents a new method, called *Ordo ab Chao*, which provides the aforementioned missing link. It complements the existing innovative thinking techniques and takes them a few steps further. It focuses on disruptive ideas creation that are tied to the existing (or emerging) disruptive technologies in order to facilitate creation of disruptive ideas with a potential for real-life implementations. Stimulated by the current COVID pandemic, it has been demonstrated how the technique can be applied in practice in higher education processes so as to make virtual lecturing comparable (or better, where possible) to face-to-face lecturing conditions. The presented Orde ab Chao application also introduces a new “disruptive direction,” which is a personalized study and training. This will entail additional educational and research standards for lecturers. If supported with appropriate technology changes, they are achievable. Contrary to common belief that cutting costs with technology provides grounds also for higher education system, we believe that this is one of core sectors for prosperity and well-being of every society, just like the healthcare sector. So, the costs may also rise. However, if technology is used accordingly to increase value added (in a disruptive way), the benefits may far exceed the costs. The *Ordo ab Chao* technique represents a tool for systematic development of such disruptive solutions, building on a creative synergy between cutting-edge technologies and innovation management approaches.

As to the evaluation of ideas developed by the presented method, we introduced the criteria of originality and potential benefits for users. Of course, for practical use in companies, additional criteria will include market analysis, cost–benefit analysis, capacity for implementation, time to market, intellectual property aspects, competition analysis, and others.

Finally, the Orde ab chao method can be positioned within a more generalized theoretical frame. For the starting point, we used a concept of three different methodological approaches to case research: theory generation, theory testing, and theory elaboration (Ketokivi and Choi, 2014). Following the criteria presented, the case research decision tree (Ketokivi and Choi, 2014), and results presented in this paper, it follows that the Orde ab Chao method fits into theory testing model. Most importantly, it is a promising tool for disruptive idea creation, which is among the most valuable “diamonds” in all organizations and professions, especially if they have the potential to become a real innovation.

## Data Availability Statement

The original contributions presented in the study are included in the article/supplementary material, further inquiries can be directed to the corresponding author/s.

## Author Contributions

BL has contributed to the research on existing innovations creation techniques and their analysis (weaknesses) with relation to disruptive technologies. DT has contributed to the analysis of Industry 4.0 disruptive technologies with focus on business views (including their business models potential), BL and DT have both contributed to the creation of the new technique based on the above inputs, its refinement, including inclusion of their own experiences with distance lecturing at uni-level during recent months (due to Corona pandemics). Both authors contributed to the article and approved the submitted version.

## Conflict of Interest

The authors declare that the research was conducted in the absence of any commercial or financial relationships that could be construed as a potential conflict of interest.

## References

[B1] Belak J. Duh M. Štrukelj T. (2017). “The higher education in Slovenia,” in *The Acceleration of Development of Transversal Competences*, Vol. 2017 eds Szafranski V. M. Golinski M. Simi H. (Kokkola: Centria University of Applied Sciences).

[B2] Bevins F. Bryant J. Krishnan C. Law J. (2020). *Coronavirus: How Should US Higher Education Plan for an Uncertain Future?.* Available online at: https://www.mckinsey.com/industries/public-sector/our-insights/coronavirus-how-should-us-higher-education-plan-for-an-uncertain-future# (accessed April 03, 2020).

[B3] Carmody L. E. (2009). Clayton M. Christensen, Michael B. Horn, and Curtis W. Johnson: disrupting class: how disruptive innovation will change the way the world learns. *Educ. Technol. Res. Dev.* 57 267–269. 10.1007/s11423-009-9113-1

[B4] Chen C. C. Chuang M. C. (2008). Integrating the Kano model into a robust design approach to enhance customer satisfaction with product design. *Int. J. Prod. Econ.* 114 667–681. 10.1016/j.ijpe.2008.02.015

[B5] Christensen C. M. (1997). *The Innovator’s Dilemma: When New Technologies Cause Great Firms to Fail.* Boston, MA: Harvard Business School Press.

[B6] Christensen C. M. Horn M. B. Johnson C. W. (2011). *Disrupting Class: How Disruptive Innovation will Change the Way the World Learns*, Vol. 1 New York, NY: McGraw-Hill.

[B7] Christensen C. M. Raynor M. E. McDonald R. (2015). What is disruptive innovation. *Harv. Bus. Rev.* 93 44–53.

[B8] Correa C. H. De Moura Ferreira Danilevicz Â. (2015). “Method for decision making in the management of innovation: criteria for the evaluation of ideas,” in *Paper Presented at the International Association for Management of Technology*, 2151–2169. Cape Town.

[B9] de Baca M. E. Arnaout R. Brodsky V. Birdsong G. G. (2015). Ordo ab chao: framework for an integrated disease report. *Arch. Pathol. Lab. Med.* 139 165–170. 10.5858/arpa.2013-0561-cp 25611099

[B10] De Bono E. Zimbalist E. (1970). *Lateral Thinking.* London: Penguin, 1–32.

[B11] Dean D. L. Hender G. M. Rogers T. L. Santanen E. L. (2005). Identifying quality, novel, and creative ideas’. *J. Assoc. Inf. Syst.* 7:30.

[B12] Ding X. Tang Y. Y. Deng Y. Tang R. Posner M. I. (2014). Mood and personality predict improvement in creativity due to meditation training. *Learn. Individ. Differ.* 37 217–221. 10.1016/j.lindif.2014.11.019

[B13] Dixon J. (1979). Quality versus quantity: the need to control for the fluency factor in originality scores from the Torrance tests. *J. Educ. Gift.* 2 70–79.

[B14] Elliot J. (1991). *Action Research for Educational Change.* Buckingham: Open University Press.

[B15] Eng J. L. Baliski C. R. McGahan C. Cai E. (2018). Uptake and impact of synoptic reporting in a community care setting. *Am. J. Surg.* 215 857–861. 10.1016/j.amjsurg.2018.01.007 29496204

[B16] Gagliordi N. (2018). *IoT to Drive Growth in Connected Devices Through 2022: Cisco. ZDNet, 27 November 2018.* Available online at: https://www.zdnet.com/article/iot-to-drive-growth-in-connected-devices-through-2022-cisco/ (accessed May 11, 2020).

[B17] IoT (2020). *IoT Glossary: 55 Terms**You Need to Know.* Available online at: https://dzone.com/articles/iot-glossary-terms-you-need-to-know (accessed July 15, 2020).

[B18] Ketokivi M. Choi T. (2014). Renaissance of case research as a scientific method. *J. Oper. Manag.* 32 232–240. 10.1016/j.jom.2014.03.004

[B19] Koshy E. Koshy V. Waterman H. (2010). *Action Research in Healthcare.* London: Sage.

[B20] Košmrlj K. Širok K. Likar B. (2015). *The Art of Managing Innovation Problems and Opportunities.* Koper: Faculty of Management.

[B21] Kudrowitz B. M. Wallace D. (2013). Assessing the quality of ideas from prolific, early-stage product ideation. *J. Eng. Des.* 24 120–139. 10.1080/09544828.2012.676633

[B22] Likar B. (2007). *Managing Innovation and R&D Processes in EU Environment.* Ljubljana: Korona plus - Institute for Innovation and Technology.

[B23] Likar B. Križaj D. Fatur P. (2006). *Management Inoviranja (EN: Innovation Management).* Koper: University of Primorska - Fakulteta za management (EN: University of Primorska - Faculty of management).

[B24] Mikulić J. Prebežac D. (2011). A critical review of techniques for classifying quality attributes in the Kano model. *Manag. Serv. Qual. Int. J.* 21 46–66. 10.1108/09604521111100243

[B25] OECD (2010). *Innovation Strategy 2010, Defining Innovation.* Available online at: https://www.oecd.org/site/innovationstrategy/defininginnovation.htm (accessed November 12, 2020).

[B26] Pečjak V. (2001). *Poti do Novih Idej*. *New Moment–Idea Book.* (Ljubljana, SLovenia: New Momemnt), 16.

[B27] Putri S. A. Muchlis M. Pujiraharjo Y. Nurfita R. (2019). “Morphological forced connection method application in the development of Plered Ceramic design,” in *Proceedings of the 6th Bandung Creative Movement 2019*, (Bandung: Telkom University), 207–211.

[B28] Reason P. Bradbury H. (2008). *The SAGE Handbook of Action Research: Participative Inquiry and Practice*, 2nd Edn London: SAGE.

[B29] Robinson K. (2010). *RSA Animate: Changing Education Paradigms.* London: The Royal Society of Arts.

[B30] Schmidt G. M. Druehl C. T. (2008). When is a disruptive innovation disruptive? *J. Prod. Innov. Manag.* 25 347–369. 10.1111/j.1540-5885.2008.00306.x

[B31] Simpson D. (2020). *The Ultimate Extended Reality (XR) Glossary.* Available online at: https://www.tvrlp.com/ultimate-extended-reality-glossary/ (accessed June 28, 2018).

[B32] Tang M. (2019). Fostering creativity in intercultural and interdisciplinary teams: the VICTORY model. *Front. Psychol.* 10:2020. 10.3389/fpsyg.2019.02020 31543855 PMC6739593

[B33] UNESCO (2020). *COVID-19 Educational Disruption and Response.* Available online at: https://reliefweb.int/sites/reliefweb.int/files/resources/en.unesco.org-COVID-19%20Educational%20Disruption%20and%20Response.pdf (accessed March 4, 2020).

[B34] Urquhart R. Porter G. A. Grunfeld E. Sargeant J. (2012). Exploring the interpersonal-, organization-, and system-level factors that influence the implementation and use of an innovation-synoptic reporting-in cancer care. *Implement. Sci.* 7:12. 10.1186/1748-5908-7-12 PMC3307439 22380718

[B35] Wiki (2020). Available online at: https://en.wikipedia.org/wiki/Industrial_Revolution (accessed July 15, 2020).

[B36] Yang C. C. Yang K. J. (2011). An integrated model of value creation based on the refined Kano’s model and the blue ocean strategy. *Total Qual. Manag. Bus. Excell.* 22 925–940. 10.1080/14783363.2011.611358

[B37] Zendejas G. Chiasson M. (2008). “Reassembling the information technology innovation process: an actor network theory method for managing the initiation, production, and diffusion of innovations,” in *IFIP International Federation for Information Processing: Open IT-Based Innovation: Moving Towards Cooperative IT Transfer and Knowledge Diffusion*, Vol. 287 eds León G. Bernardos A. Casar J. Kautz K. DeGross J. (Boston, MA: Springer), 527–539. 10.1007/978-0-387-87503-3_30

